# Identification of non-cerebral cyst: Zoonotic *Taenia multiceps* in domestic goat in Bangladesh

**DOI:** 10.14202/vetworld.2017.1156-1160

**Published:** 2017-10-01

**Authors:** Mohammad Omer Faruk, A. M. A. M. Zonaed Siddiki, Md. Shafiqul Islam, Azizunnesa Rekha, Sharmin Chowdhury, Md. Masuduzzaman, Mohammad Alamgir Hossain

**Affiliations:** 1 Department of Pathology and Parasitology, Faculty of Veterinary Medicine, Chittagong Veterinary and Animal Sciences University, Khulshi 4225, Chittagong, Bangladesh; 2 Department of Medicine and Surgery, Faculty of Veterinary Medicine, Chittagong Veterinary and Animal Sciences University, Khulshi 4225, Chittagong, Bangladesh

**Keywords:** 12SrRNA, Bangladesh, goat, non-cerebral cyst, phylogeny, *Taenia multiceps*

## Abstract

**Aim:**

This study was performed to identify the non-cerebral *Taenia multiceps* cyst through molecular phylogeny of the 12S rRNA gene.

**Materials and Methods:**

Eight cyst samples were collected from 385 examined slaughtered goats during October 2015-September 2016 from three slaughterhouses in Chittagong City Corporation. Cysts were removed from the thigh muscle, and scolices were collected for light microscopic examination and molecular identification. The DNA was extracted and analyzed by polymerase chain reaction using 12S rRNA gene primers. Cyst samples were also preserved in 10% buffered formalin for histopathological study.

**Results:**

*T. multiceps* non-cerebral cyst is 2.1% prevalent in goat in this area. Under light microscopic examination, scolex was found with four suckers and a rostellum with the double crown of 32 hooks and hooklets. Molecularly, all the samples were amplified with 12S rRNA gene fragments yielded 270 base pair amplicon. Zenker’s necrosis with focal to diffuse infiltration of lymphocytes and eosinophil was also found around the cyst wall in histopathological examination.

**Conclusion:**

Although the non-cerebral form of the cysts produced by *T. multiceps* is genetically identical with the cerebral cyst, previously published data indicated that cerebral *T. multiceps* cyst is predominant in other parts of the world as well as in Bangladesh. This study showed that non-cerebral cyst is also prevalent in this country which is very important for public health concern. This study depicts an idea of non-cerebral form of zoonotic *T. multiceps* cyst which will be helpful in taenia cyst control and prevention.

## Introduction

The cestode *Taenia multiceps* is a causative agents for coenurosis in sheep and goat affect the central nervous system (CNS) particularly the brain of sheep and goats but rarely in the spinal cord also called *Coenurus cerebralis* [1,2]. Coenurosis is widely recognized as zoonotic parasite [3] and is presents as an endemic condition in small ruminant in Bangladesh [4]. The disease is recorded as 2.4-5.6% in goat in Bangladesh [5,6]. Environmental factors such as rainfall, relative humidity, and air temperature are considered to be the risk factors for coenurosis in this country [7].

Besides this, a lack or limited use of anticestodal drug, uncontrolled backyard slaughtering, and improper offal disposal are also responsible for transmission of this cestode [8]. The cerebral form of the cysts produced by *T. multiceps* in sheep is genetically identical with the non-cerebral cysts of *T. multiceps gaigeri* [9,10] in intramuscular and/or subcutaneous tissues in goats [11,12]. Recent molecular phylogenetic analysis of nad1, cox1, and 12S rRNA showed that *T. multiceps* can cause cerebral coenurosis in sheep and non-cerebral forms in goats but rarely in sheep [13]. Information on intramuscular and subcutaneous (other organs) coenurosis in goats is limited and incomplete. Molecular study of *T. multiceps* has not been done yet, but *Echinococcus granulosus* has been identified molecularly in cattle in Bangladesh [14].

The aim of the present study was to determine the prevalence, morphological features of parasite scolex, and molecular detection of *T. multiceps* besides phylogenetic analysis of the goat isolates sequence with NCBI reference sequences for characterization of cyst in this area.

## Materials and Methods

### Ethical approval

All samples were collected and complies with relevant legislation. It follows the international guiding principles for biomedical research involving animals.

### Collection of *T. multiceps* cysts

Eight cyst samples were collected from 385 examined slaughtered goats in three slaughterhouses located in Chittagong City Corporation during October 2015-September 2016. After gross examination cysts were removed from the parasitized organs and preserved in 10% buffered formalin for histopathological study and the scoleces or larval tissue material from cysts was preserved in 90% ethanol for molecular study. Scolices were collected through scraping from the sides of germinal layer of the cyst also stained with eosin stain for light microscopic examination.

### Genomic DNA extraction and amplification by polymerase chain reaction (PCR)

Genomic DNA was extracted from protoscolices using iNtRoN Patho gene Spin DNA/RNA Extraction Kit according to the manufacturer’s recommendations. Genomic DNA concentrations were determined spectrophotometrically by Qubit 2.0 (Invitrogen, Korea), and the DNA samples were stored at −20°C until used. 12S rRNA gene primers were designed according to the mitochondrial genome sequence of *T. multiceps* as follows: Tae-F (5’-GATTCTTTTTAGGGGAAG G-3’) and Tae-R (5’-GCGGTGTGTACMTGAGCTAAAC-3’). 50 µl of PCR reaction was performed using 10 mM Tris-HCl, 50 mM KCl, 1.5 mM MgCl_2_, 0.2 mM of each deoxynucleotide triphosphate, 20 pmol of each primer, 100 ng template gDNA, and 2 U *Taq* DNA polymerase (Tokyo, Japan) in each reaction under the following cycling conditions: After an initial denaturation at 94°C for 5 min, then 94°C for 30 s (denaturation), 55°C for 1 min (annealing), and 72°C for 30 s (extension) for 35 cycles, followed by a final extension at 72°C for 10 min. Samples without genomic DNA were included in each amplification run as negative controls. PCR products were separated on a 1% agarose gel and detected by ethidium bromide staining.

### Sequencing and phylogenetic analysis

Four PCR products were purified by PCR purification kit (Favor Prep™ PCR Clean-Up Mini kit, Favorgen Biotech Corporation, www.favorgen.com) according to the manufacturer’s instructions, and then, these purified products send to Bioneer, Korea, for sequencing. Sequence chromatograms were analyzed using the Chromas Pro 2 (South Brisbane and Australia) and Mega 6.0 computer software programs [15]. Available reference sequence of *T. multiceps* was retrieved from NCBI GenBank, and *E. granulosus* (as outgroup) also inferred from other publications [16]. Sequences were compared with each other using Chromas and BLASTn program. After multiple alignments by ClustalW, phylogenetic analyses of the sequences data were performed using 12S rRNA gene sequences, and phylogeny tree was drawn using sequences obtained in this study as well as reference sequences of all described *T. multiceps* by MEGA6 software.

### Histopathology

Tissue specimens collected from the affected muscular area were fixed in 10% neutral buffered formalin. The paraffin blocks were prepared, and sections of 4-5 µm thickness were obtained on glass slides with a rotatory microtome. These paraffin sections were stained with hematoxylin and eosin stain for routine histopathology [17].

## Results

**Figure-1:**
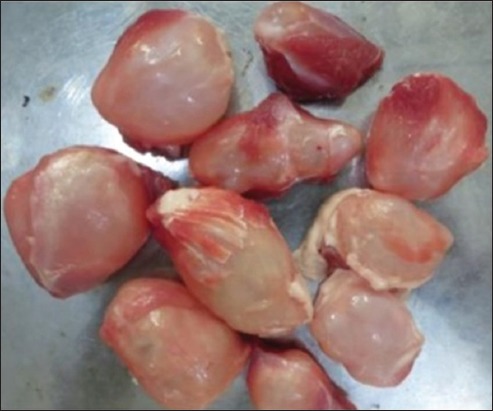
Collected cyst from thigh muscle.

In this study, a total of 385 domestic goats were examined during postmortem. Eight animals (2.1%) were found infected with taeniid cysts with an average diameter of 2.7 cm (Table-1). The entire cysts were observed in the thigh muscle, inguinal region, and in the prescapular region (Figure-1).

**Table-1:** Occurrence of *Taenia* spp. cyst.

Species	Number of examined	Number of infected animal	Prevalence %	Total number of cyst	Average diameter of the cyst (cm)	Mean or average number of cyst/animal
Goat	385	8	2.1	42	2.7	5.25

**Figure-2:**
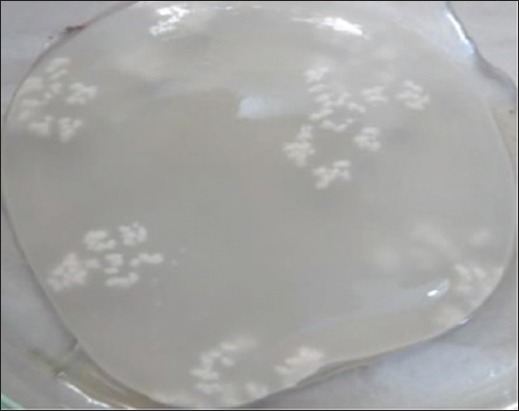
White clusters indicating a large number of scolices attached to the internal layer of cyst.

**Figure-3:**
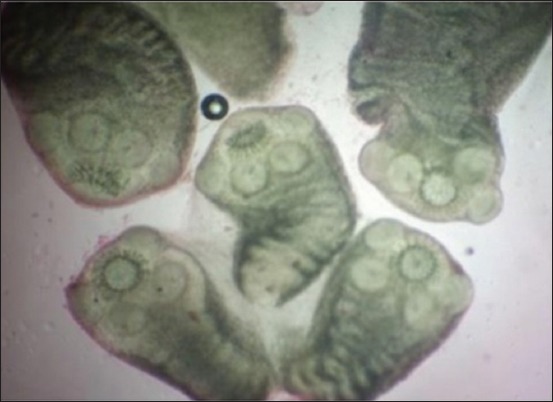
Larvae of *Taenia multiceps* multiple scolex (10×).

**Figure-4:**
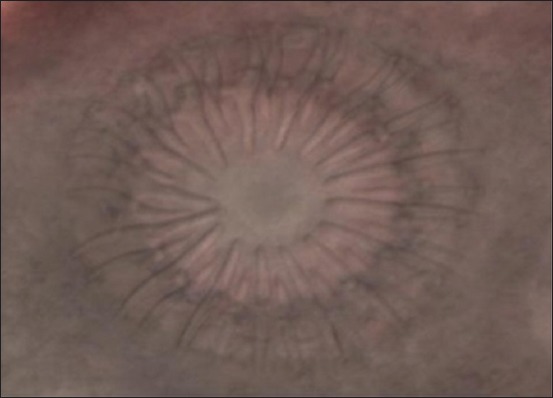
Double layer of rosteller hook.

The characteristics morphology of *T. multiceps* larvae was seen in all of the cysts containing clear fluid with numerous fertile protoscolices. A large number of scolices as white clusters were attached to the internal cyst wall (Figure-2). Under the microscope, larvae or scolex was examined at 10× magnification (Figure-3). Most of the scolices had four suckers and a rostellum with a double crown of 32 hooks with hooklets (Figure-4).

### Histopathological examination of the cyst

**Figure-5:**
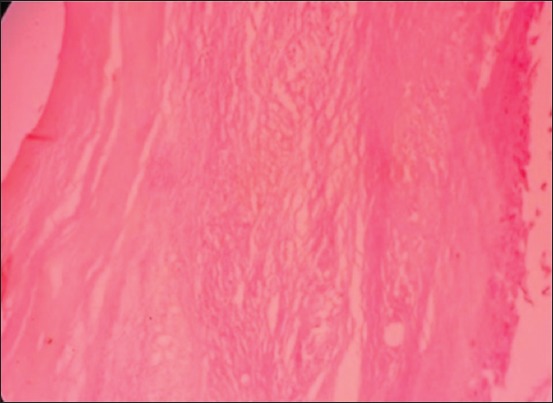
Thin layer of fibrous cyst walled and atrophy of adjacent muscle bundle.

**Figure-6:**
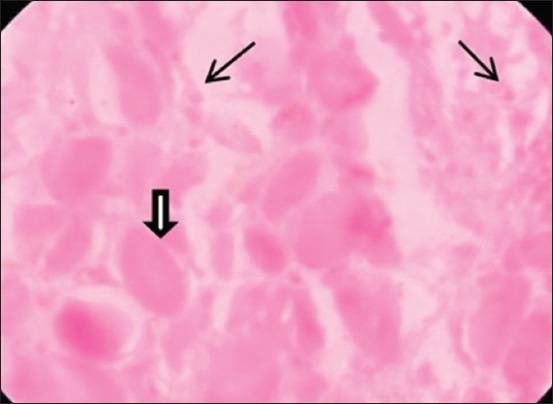
Infiltration of reactive cell (thin arrow) and Zenker’s necrosis (thick arrow).

The thin fibrous cyst wall causes mechanical destruction in muscle layer associated with degenerative and necrotic changes including hyaline degeneration and Zenker’s necrosis (Figure-5). Diffuse infiltration of lymphocytes and eosinophil was also found around the cyst wall and between muscle fibers (Figure-6).

### Molecular study and phylogenetic analysis

**Figure-7:**
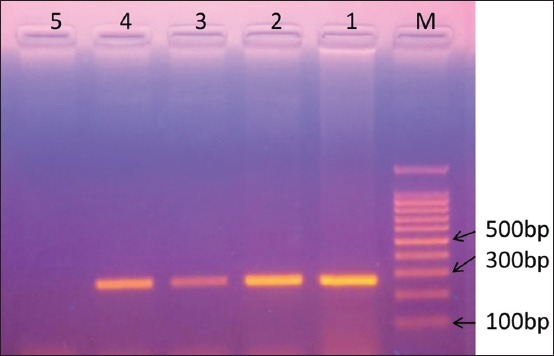
Agarose gel electrophoresis of polymerase chain reaction amplified 270 bp amplicons of 12S rRNA gene fragments. The Lane M indicates 100 bp ladder; S1-S4 positive sample; and S5 negative DNA sample.

**Figure-8:**
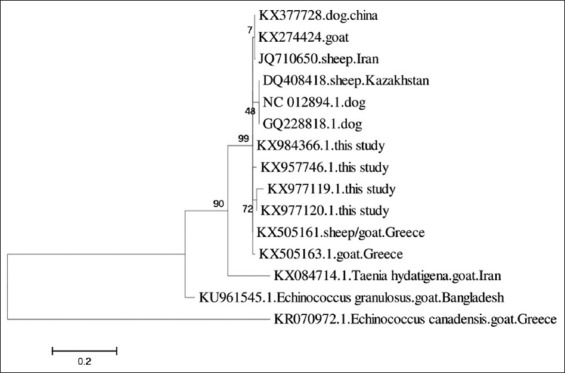
Phenogram constructed for the *Taenia multiceps* isolates of Chittagong, Bangladesh, and reference sequence with complete mitochondrial 12S rRNA gene sequence (GQ228818) using maximum likelihood method at 1500 bootstrap values. *Echinococcus* spp. and *Taenia hydatigena* were drawn as an outer group.

Eight samples were amplified with 12S rRNA gene fragments yielded 270 base pair amplicon (Figure-7). These samples were identified as *T. multiceps*. Four sequences were submitted to the GenBank under following accession numbers - KX977119, KX977120, KX957746, and KX984366. Phylogenetic tree constructed through using the sequence data indicates that all the isolates from the present study were grouped into a distinct cluster of sheep and goat originated *T. multiceps* sequences (Figure-8).

## Discussion

Coenurosis is an endemic disease of Bangladesh although very few studies have been conducted in this country and all of them are only epidemiological or case study [4]. The present study was carried to determine the prevalence of *T. multiceps* in goat with their molecular detection and characterization. A total of 385 goats were examined postmortem, and 2.1% prevalence was recorded. All of the examined cysts were found in muscle and subcutaneous tissue of slaughtered goat. This is the first molecular study of the non-cerebral form of *T*. *multiceps* in goats in the country. Although the cerebral form of *T. multiceps* (*C. cerebralis*) in goats was reported in some area, 2.5% at Patuakhali district [18] and 35 cases found in Mymensingh district during the period 2010-2011 [19]. Coenurosis in calves is also reported as 2.47% in Bangladesh [20]. In comparison with this study, *C. cerebralis* cyst is found in the thigh muscle [21] and subcutaneous tissue [22] of goat in Uttar Pradesh, India. In another study in Southern Iran, it is found that same parasite produced both cerebral and non-cerebral forms in sheep and goats [23]. In sheep, the common predilection sites of these parasites are the CNS. The coenurus cyst that occurred outside the CNS in goats was recorded as *T. multiceps* [2,24] or *T. gaigeri* [25]. Genetically, the cerebral cysts in sheep were identical with the non-cerebral cysts in goats [10]. Histopathological findings in the skeletal muscle infected with coenurus included degenerative and necrotic changes, hyalinization, and myositis which are in agreement with other research [11,26].

Although coenurosis particularly in cerebral form in goat was reported in different area of Bangladesh, the molecular identification of *T. multiceps* was not done yet. Previously reported studies in other countries were based on amplification of several genes such as 12S rRNA gene, cox1 gene, and nad1 gene. We used only 12S rRNA gene marker. The present molecular genetic approach was employed to characterize the DNA sequence of the non-cerebral metacestodal stage of *T. multiceps* and compared it with other available data from other countries. Goat non-cerebral thigh muscle cyst isolated sequences of this study were aligned with Greece sheep and goat, Iranian sheep, and complete 12S rRNA gene of China.

## Conclusions

This study showed that non-cerebral cyst is prevalent in this country which is very important for public health concern. This study depicts an idea of the non-cerebral form of zoonotic *T. multiceps* cyst which will be helpful in taenia cyst control and prevention.

## Authors’ Contributions

Research work was done by MOF and partly MSI. Research design was carried out by AMAMZS, MAH, and SC. All the authors participated in data analysis, while MM, AR, and MSI revised the manuscript. All authors read and approved the final manuscript.
